# Chitinase Gene *FoChi20* in *Fusarium oxysporum* Reduces Its Pathogenicity and Improves Disease Resistance in Cotton

**DOI:** 10.3390/ijms25158517

**Published:** 2024-08-04

**Authors:** Hui Lou, Jincheng Zhu, Zengqiang Zhao, Zegang Han, Wei Zhang

**Affiliations:** 1 The Key Laboratory of Oasis Eco-Agriculture, Agriculture College, Shihezi University, Shihezi 832000, China; louhui@stu.shzu.edu.cn (H.L.); nkyzjc1998@163.com (J.Z.); tlx4109@126.com (Z.Z.); 2 Zhejiang Provincial Key Laboratory of Crop Genetic Resources, the Advanced Seed Institute, Plant Precision Breeding Academy, College of Agriculture and Biotechnology, Zhejiang University, Hangzhou 310000, China

**Keywords:** cotton, root secretion, *Fusarium oxysporum*, chitinase genes, *Fusarium wilt*

## Abstract

Chitinase genes, as a class of cell wall hydrolases, are essential for the development and pathogenesis of *Fusarium oxysporum* f.sp. *vasinfectum* (*F. ox*) in cotton, but related research focused on chitinase genes are limited. This study explored two island cotton root secretions from the highly resistant cultivar Xinhai 41 and sensitive cultivar Xinhai 14 to investigate their interaction with *F. ox* by a weighted correlation network analysis (WGCNA). As a result, two modules that related to the fungal pathogenicity emerged. Additionally, a total of twenty-five chitinase genes were identified. Finally, host-induced gene silencing (HIGS) of *FoChi20* was conducted, and the cotton plants showed noticeably milder disease with a significantly lower disease index than the control. This study illuminated that chitinase genes play crucial roles in the pathogenicity of cotton wilt fungi, and the *FoChi20* gene could participate in the pathogenesis of *F. ox* and host–pathogen interactions, which establishes a theoretical framework for disease control in Sea Island cotton.

## 1. Introduction

Due to the superior fiber quality, island cotton (*Gossypium barbadense*) serves as an essential material for high-quality and special textiles all over the world [1,2,3,4,5]. Xinjiang is the only place in China that produces island cotton. However, numerous biotic and abiotic stresses frequently have negative impacts on cotton quality and output [6,7,8,9,10].

*Fusarium oxysporum* f. sp. *vasinfectum* (*F. ox*), one of the most dangerous quarantine diseases in cotton production, is a widespread soilborne disease that affects cotton plants, decreasing the cotton yield and fiber quality [11,12,13,14,15]. A Hemipteran subphylum called Fusarium produces conidia on the lateral spore peduncle through hyaline, segregating hyphae [16]. Fusarium wilt, caused by *F. ox*, frequently results in dead plants in the cotton seedling and bud stage. The quality and quantity of the remaining bolls on the diseased plants are reduced additionally, which is harmful for cotton production and the income of cotton farmers [17,18,19,20,21,22]. With the spread of *F. ox*, it has gradually threatened the development of the island cotton in Xinjiang, which is more sensitive to this disease generally.

Cotton root secretion is closely related to the pathogenicity of *F. ox* because the pathogen typically invades cotton from the root tip or root wound, where it produces conidia, expands upward, and is triggered to germinate by cotton root secretion [23,24,25,26,27]. Root secretions from most of the pea germplasms stimulated *F. ox* germination, but root secretions from three germplasms inhibited *F. ox* germination. The content of the active substance Pisatin in the root secretions was negatively correlated with *F. ox* germination, suggesting that root secretions play important roles in the growth of *F. ox* [28]. Coincidentally, watermelon wilt was substantially less common when watermelon was intercropped with wheat because the root secretions from the wheat encouraged the growth of the watermelon roots and induced physiological and genetic changes that made the fruit resistant to the disease [29,30]. Similarly, Yang et al. confirmed that intercropping wheat and fava beans raised the concentrations of serine, glutamate, tyrosine, and lysine in the root secretions of the fava bean, which prevented *F. ox* growth and reproduction and decreased Fusarium wilt [31]. Moreover, the production per plant increased and the pathogenicity index of the cucumber wilt disease decreased by using the onion cucumber intercropping planting method [32].

Chitin, glucans, and polysaccharides make up the majority of the complex structures that contribute to the cell walls of filamentous fungi [33,34,35,36,37]. The hydrolases known as chitinases accelerate the breakdown of the 1,4-bond in chitin, releasing N-acetylglucosamine oligomers as a result. The processes of spore germination, tip growth and mycelial branching, spore differentiation, autolysis, and parasitization are hypothesized to be affected by chitinases in filamentous fungi [38,39,40,41]. *PstChia1* had chitinase activity and was highly stimulated early in the contacts between wheat-stripe rust, and the silencing of *PstChia1* suggested that *PstChia1* restricted *Pst* growth while decreasing *Pst* virulence, suggesting that *PstChia1* is involved in the control of stripe rust resistance in wheat [42]. In recent years, extensive research has shown that phytopathogenic fungi can use self-secreted chitinases to evade chitin-triggered plant immunity [43,44,45]. The extracellular chitinase *MoChi1* of *Magnaporthe oryzae* can competitively bind chitin and inhibit chitin-triggered plant immunity [43]. PbChia1 protein can degrade dormant spores of *Rhizoctonia solani* in vitro and inhibit the germination. Treating hydroponically grown oilseed rape rhizoctonia with this protein significantly reduced the content of *R. solani* in the roots, delayed the development of *R. solani*, and alleviated the symptoms of rhizoctonia in oilseed rape rhizoctonia [44]. Furthermore, MoAa91, a chitin-binding protein secreted by *M. oryzae*, competes with the rice chitin receptor *OsCEBiP* to bind free chitin oligosaccharides and inhibit chitin-triggered immune responses in rice, including reactive oxygen species bursts, callose deposition, and defense-related gene expression [45].

Currently, there are primarily two methods for controlling cotton wilt: biological control and chemical control; the former involves growing resistant types and rotating crops, and the latter has issues with residue and environmental contamination [17]. Thus, it is urgently necessary to identify the genes of *F. ox* associated with pathogenicity to offer a theoretical foundation for the control of island cotton wilt disease. While the effects of root secretions from various cotton varieties on the expression of *F. ox* genes have still not been well studied, some researchers have partially uncovered the molecular mechanisms behind *F. ox* growth and pathogenicity development. In previous studies based on transcriptomic data from co-cultures of resistant and susceptible cotton varieties’ root secretions with Fusarium wilt, we have discovered the susceptible island cotton cultivar Xinhai 14 (XH-14)’s root secretion encouraged the growth of *F. ox* and the resistant cultivar Xinhai 41 (XH-41)’s root secretion hindered it; 1514 novel DEGs were found [46]. Thus, in the present study, a weighted correlation network analysis (WGCNA), a Gene Ontology (GO) analysis, and a Kyoto Encyclopedia of Genes and Genomes (KEGG) enrichment analysis were performed to figure out the differentially expressed modules. We explored their transcriptional-level interaction mechanisms and screened candidate genes involved in *F. ox* growth, development, and pathogenicity. Additionally, we defined the *FoChi* gene family on the genome-wide level of *F. ox* and investigated the role *FoChi20* plays in cotton resistance and *F. ox* pathogenicity. Our findings could establish the groundwork for investigating the molecular mechanisms behind the pathogenicity of *F. ox* and its interaction with cotton, providing a theoretical framework for future functional analyses of potential genes to improve cotton wilt control methods and making a contribution to island cotton resistance research for *F. ox*.

## 2. Results

### 2.1. Identification of Disease Resistance in Cotton and Restoration Culture

**Figure 1:**
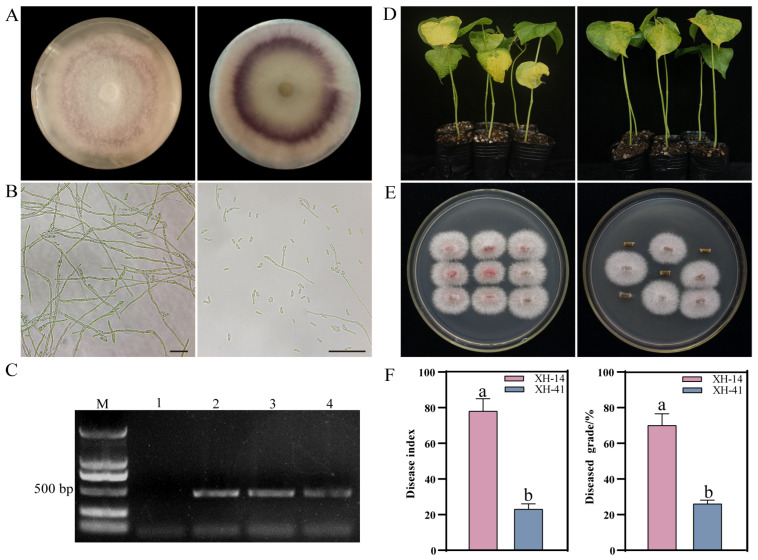
The *F. ox* resistance investigation of XH-41 and XH-14 varieties. (**A**) The front and rear sides of a strain, F327 colony, on PDA medium. (**B**) Mycelial (**left**) and conidial (**right**) morphology. Bar = 20 μm. (**C**) The PCR detection of *F. ox* using specific primers. M indicates the DNA marker. Lane 1 represents the outcome of PCR amplification using water as a template. Lanes 2, 3, and 4 represent the results of PCR amplification of the *F. ox* cDNA. (**D**) XH-14 (**left**) and XH-41 (**right**) phenotypes after 21 d of *F. ox* inoculation. (**E**) The phenotype of XH-14 (**left**) and XH-41 (**right**) stalk pathogens after 14 d inoculation with *F. ox.* (**F**) The disease index and grade statistics after 21 d of *F. ox* infection. Different letters indicate significant differences established at *p* < 0.05.

To identify the strain as *F. ox*, we isolated and cultured the strain. The colonies were filamentous, felt-like, white, light pink to slightly purple, and filamentous (Figure 1A). In Czapek’s medium, the colonies generated large and small conidia with the big conidia being unicellular and ovate and the small conidia being primarily 3-septate and falcate (Figure 1B). The donor organisms all produced bands of around 442 bp in length that were amplified using *F. ox*-specific primers and proved that the strain was *F. ox* (Figure 1C).

Furthermore, two Sea Island cotton cultivars with different resistance levels for Fusarium wilt were employed for root secretion collection. When these two cultivars were infected by F327, XH-14 displayed significant leaf wilt and premature defoliation symptoms after 21 days, but XH-41 displayed only marginal wilt symptoms (Figure 1D). We conducted the *F. ox* recovery test on the stems of inoculation-treated cotton seedlings 21 days after inoculation, revealing that XH-14 and XH-41 plants displayed various degrees of resistance to *F. ox* infestation, with XH-14 plants having much more fungal biomass in their stems than XH-41 plants (Figure 1E). The disease index post-14 days of infection for XH-14 was 78, much higher than that for XH-41, which was 23. The incidence rate post-21 days of infection for XH-14 was 70, also much higher than that for XH-41, which was 26 (Figure 1F). Thus, XH-14 and XH-41 served as susceptible and resistant materials, respectively.

### 2.2. Hub-Genes with Pathogenicity of F. ox

**Figure 2:**
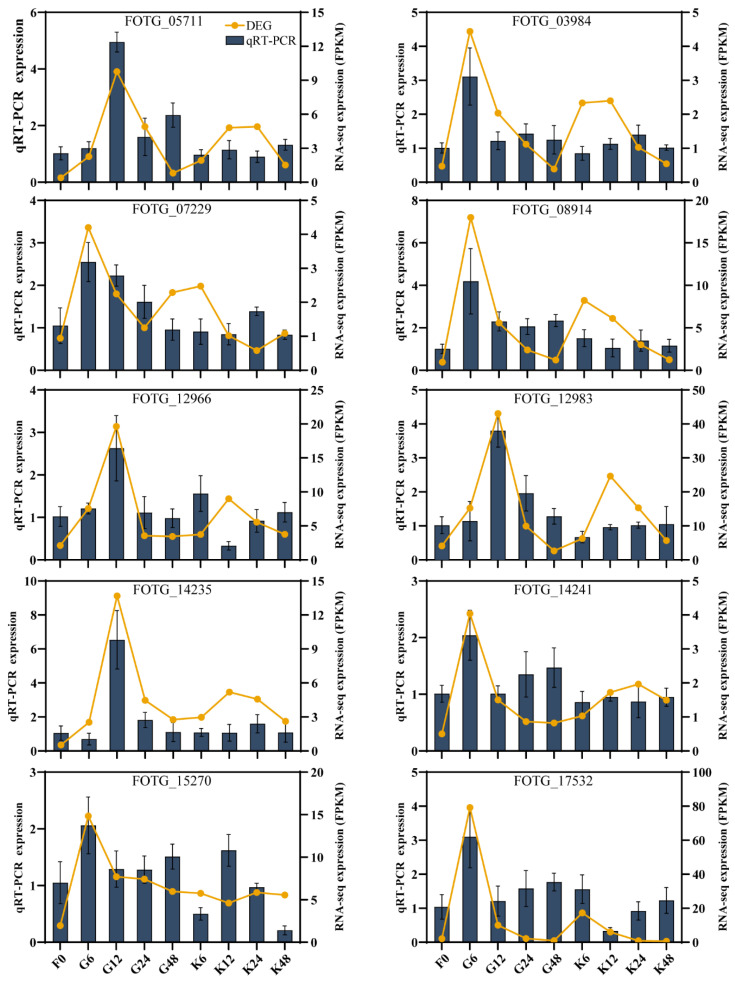
The verification of differentially expressed genes in *F. ox* by RT-qPCR. The gene ID is located in the middle of each plot. The yellow line indicates the expression level of each DEG, and the blue bar indicates the RT-qPCR expression. F represents the treatment at 0 h. G represents the susceptible root secretion treatment for 6, 12, 24, and 48 h, respectively. K represents the resistant root secretion treatment for 6, 12, 24, and 48 h.

Based on transcriptomic data obtained from our previous studies [46], ten DEGs were randomly selected for RT-qPCR, showing that the expression patterns of DEGs were highly consistent with the RNA-seq results (Figure 2).

**Figure 3:**
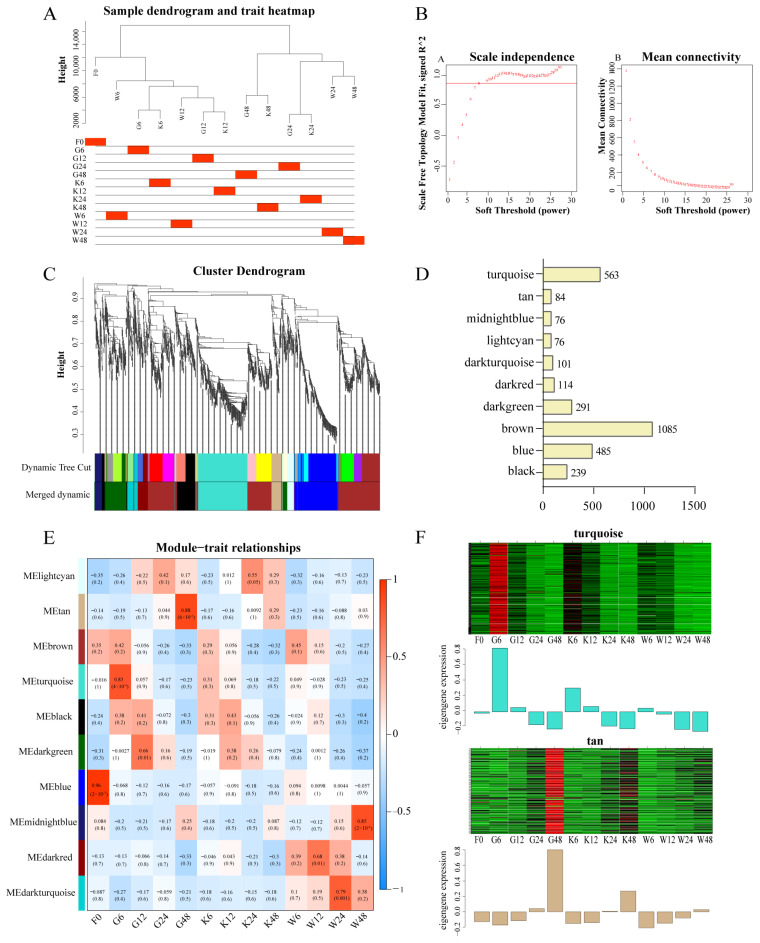
Gene co-expression network and correlation of each module with *F. ox* pathogenicity. (**A**) The clustering dendrogram of samples and tissue correspondence. (**B**) Soft threshold determination. (**C**) Gene clustering tree via modular partitioning and topological dissimilarity matrices. (**D**) The number of genes in each module. (**E**) *F. ox* pathogenicity gene modules related to the samples. Blue showed low transcription levels, while red indicates high transcription levels. (**F**) Gene expression heatmap for the tan and turquoise modules. Green denotes low transcription levels, whereas red shows high transcription levels.

In the present study, WGCNA was used to gain a thorough understanding of the gene regulatory network strongly associated with *F. ox* pathogenicity. The *F. ox* gene expression matrix was filtered based on the FPKM value to remove genes with lower expression, and 4248 DEGs were identified. The results of sample clustering and trait association of gene expression levels showed that the gene clustering tree of each treatment could correspond well with it (Figure 3A). According to the results of the soft threshold calculation, *β* = 8 was chosen to construct the network, and the dynamic shear tree method was used to merge the modules with similar expression (Figure 3B). A total of 10 co-expression modules were obtained (Figure 3C). In addition, different numbers of genes were classified in different modules. Of them, the brown module had the highest number of genes with 1085 genes, while the light cyan and midnight blue modules had the lowest number of genes at 76 (Figure 3D). Four of the modules, midnight blue, blue, turquoise, and tan, indicated the strongest pathogenic correlation with *F. ox* (Figure 3E). The turquoise (*r* = 0.83, *p* = 4 × 10^-4^) and tan (*r* = 0. 88, *p* = 6 × 10^-5^) modules were obtained by screening with a threshold of *r* > 0.70 and *p* < 0.005. In addition, the expression of DEGs was particularly high in the turquoise module co-cultivated with root secretions of a susceptible variety for 6 h and in the tan module co-cultivated with root secretions of a susceptible variety for 48 h, suggesting that the gene function of these two modules may be involved in the pathogenicity of *F. ox* (Figure 3F).

**Figure 4:**
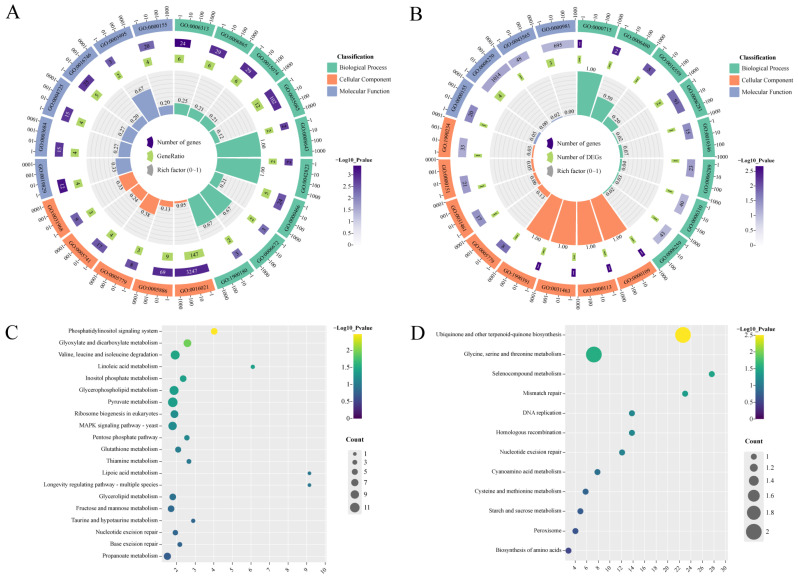
GO functional enrichment and KEGG metabolic pathway enrichment analysis of different modules. (**A**) Turquoise module GO function enrichment. (**B**) Tan module GO function enrichment. (**C**) Turquoise module KEGG metabolic pathway enrichment. (**D**) Tan module KEGG metabolic pathway enrichment.

Furthermore, GO and KEGG enrichment were conducted to identify gene function in the turquoise and tan modules. As a result, the DEGs in the turquoise module were enriched in transposition, DNA-mediated (GO:0006313), amino acid transport (GO:0006865); an integral component of membrane (GO:0016021); the plasma membrane (GO:0005886); and cation-transporting ATPase activity (GO:0019829) as well as damaged DNA binding (GO:0003684) (Figure 4A), while the DEGs in the tan module were mainly enriched in nucleotide-cleavage repair, DNA damage recognition (GO:0000715), N-terminal protein amino acid methylation (GO:0006480), the nucleotide-cleavage repair complex (GO:0000109), nucleotide-cleavage repair factor 4 complex (GO:0000113), phospho-stacking-sensing kinase activity (GO:0000155), and zinc ion binding (GO:0008270) among other processes (Figure 4B). For the KEGG enrichment analysis, the DEGs in the turquoise module largely belonged to the phosphatidylinositol signaling system; glyoxylate and dicarboxylate metabolism; valine, leucine, and isoleucine degradation; linoleic acid metabolism; inositol phosphate metabolism; etc. The DEGs in the tan module were mainly classed into ubiquinone and other terpenoid-quinone biosynthesis; glycine, serine, and threonine metabolism; selenocompound metabolism; mismatch repair; DNA replication; etc. (Figure 4C,D). In general, amino acids in the above pathway affect the formation of *F. ox* thick-wall spores and are involved in the regulation of the pathogenicity of this pathogen: key components of the phosphatidylinositol signaling system are involved in the regulation of the *F. ox* pathogenicity process. These results showed that the genes in the turquoise and tan modules may be closely related to the pathogenicity of *F. ox*.

**Figure 5:**
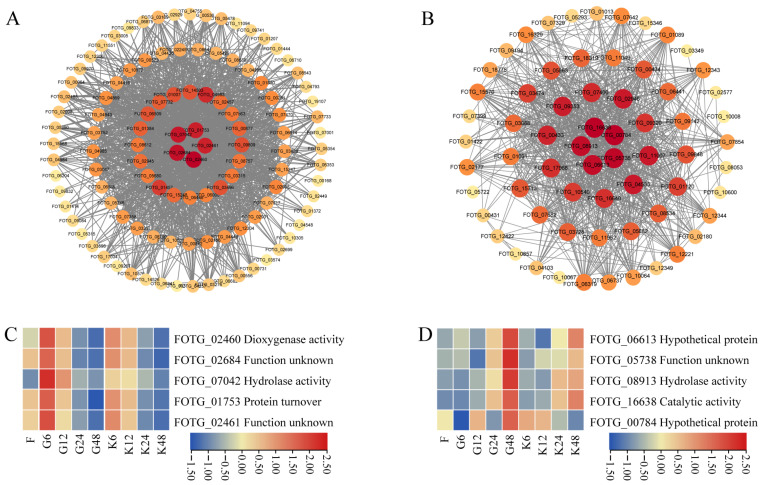
Gene expression patterns in the turquoise and tan modules. (**A**) The main gene network in the turquoise module. (**B**) The main gene network in the tan module. (**C**) The heatmap of top 5 most connected genes in the turquoise module. (**D**) The heatmap of the top 5 most connected genes in the tan module. F represents the treatment at 0 h. G represents the susceptible root secretion treatment for 6, 12, 24, and 48 h, respectively. K represents the resistant root secretion treatment for 6, 12, 24, and 48 h.

The top 5 genes with connectivity in the turquoise and tan modules were selected as core genes, and these genes and their associated genes (weight value > 0.20) were mapped in a visual gene interaction network (Figure 5A,B). In the turquoise module, there were FOTG_02460 (dioxygenase activity), FOTG_02684 (function unknown), FOTG_07042 (hydrolase activity), FOTG_01753 (protein turnover), and FOTG_02461 (function unknown). In the tan module, there were FOTG_06613 (hypothetical protein), FOTG_05783 (function unknown), FOTG_08913 (hydrolase activity), FOTG_16638 (catalytic activity), and FOTG_00784 (hypothetical protein). The expression of these genes was examined, and all five genes of the turquoise module showed a significant increase in gene expression at 6 h of co-cultivation of root secretions from the susceptible variety (XH-14), whereas gene expression was lower at 24 and 48 h of co-cultivation of root secretions from the susceptible variety (Figure 5C). All five genes in the tan module showed significantly higher gene expression at 48 h of root secretion co-cultivation of the susceptible variety and lower gene expression at 6 h and 12 h of root secretion co-cultivation of the susceptible variety (Figure 5D). Among them, the gene FOTG_07042, which is related to the activity of *F. ox* lipase, was significantly higher in the root secretion co-culture of the susceptible variety at 6 h than the other time treatments, indicating that it might be closely related to the pathogenicity of *F. ox*, and therefore, this candidate gene was selected for further study and named *FoChi20*.

### 2.3. FoChi Genes in F. ox Were Involved in Resistant and Susceptible Cotton Root Secretions

Considering the irreplaceable roles of chitinase genes in Fusarium wilt pathogenicity and plant resistance response, we first identified the chitinase genes on the whole genome of *F. ox*. A total of 25 chitinase genes, named *FoChi1-FoChi25*, were identified in the *Fusarium oxysporum* genome. The coding sequence (CDS) length of these chitinase genes ranged from 444 bp to 4005 bp, encoding 147 to 1334 amino acids, respectively. The protein molecular weight of the protein ranged from 15.80 to 145.83 kD correspondingly. The minimum theoretical isoelectric point was 4.55, and the maximum was 9.16. The hydrophobicity index ranged from −0.722 to −0.049, showing hydrophilicity. Fifteen genes were predicted to be localized to extracellular space, while *FoChi9*, *FoChi11*, *FoChi21*, *FoChi1*, *FoChi25*, *FoChi7*, *FoChi24*, *FoChi2*, *FoChi4*, and *FoChi13* were localized to the cytoplasm, endoplasmic reticulum., mitochondria, nucleus, and peroxisome (Table S2).

**Figure 6:**
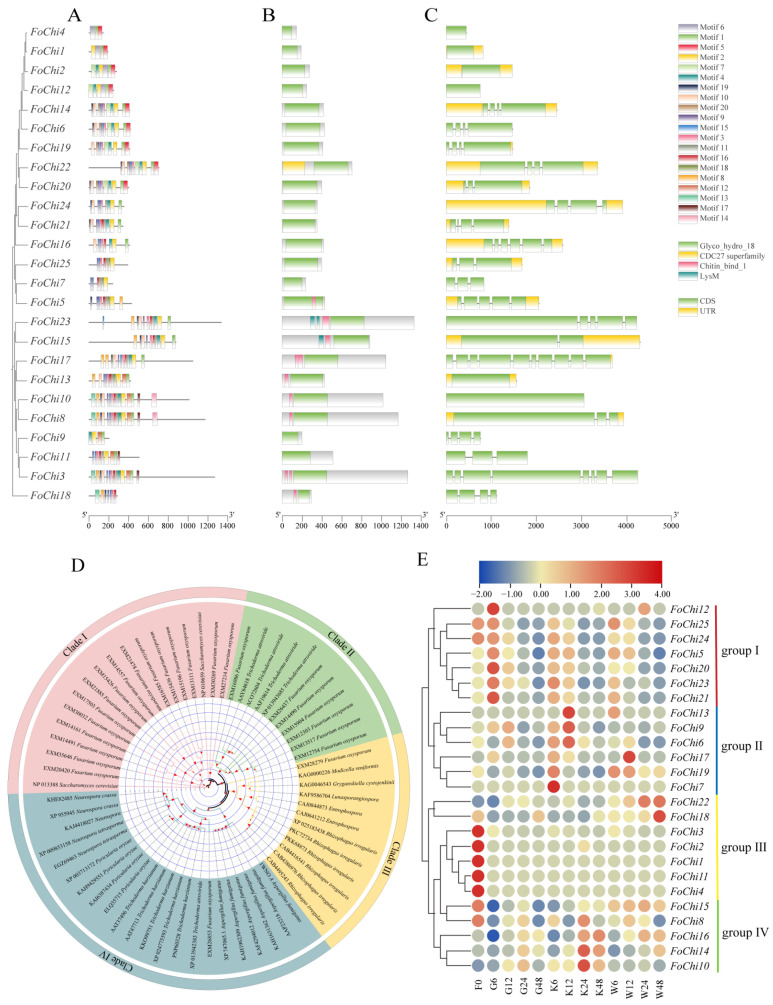
Information for the characteristics of *F. ox* genes. (**A**) The motifs of the FoChi protein. The protein sequence and conserved motifs were indicated by black lines and different colored boxes, respectively. (**B**) The structural domains of the *F. ox FoChi* gene. (**C**) Exon–intron structures of the 25 *FoChi* genes of *F. ox*. The yellow boxes, green boxes, and black lines indicated untranslated regions, exons, and introns, respectively. (**D**) The expression profiling of *FoChi* genes after co-culture with roots. (**E**) The evolutionary tree analysis of *FoChi* gene-encoded proteins.

Further, there were a total of 18 conserved motifs in the *FoChi* genes. Apart from *FoChi4* and *FoChi18*, the remaining 23 *FoChi* genes contain motif 2. The conserved motif 1, motif 2, and motif 6 were present in *FoChi1*, *FoChi2*, and *FoChi12*. With 15 motifs each, *FoChi8* and *FoChi10* contained the greatest number of motifs; all of their motifs were of the same type and arranged in the same sequence. *FoChi4* had the fewest motifs with only three (Figure 6A). All 25 chitinase genes contained the Glyco_hydro_18 structural domain, which involved hydrolyzed glycosidic bonds and belonged to the glycoside hydrolase 18 family. A total of nine genes, *FoChi5*, *FoChi23*, *FoChi15*, *FoChi17*, *FoChi13*, *FoChi10*, *FoChi8*, *FoChi3*, and *FoChi18*, contained the Chitin_bind_1 structural domain, and the *FoChi23* and *FoChi15* genes also contained LysM (Figure 6B). Chitin_bind_1’s structural domain was involved in the recognition and binding of chitin, and LysM was mainly involved in chitin synthesis in eukaryotes.

Exons and introns counted for 25 *FoChi* genes and ranged from 0 to 9. Sixteen *FoChi* genes contained 2~5 exons, three included 6~8 exons, and six genes had just 1 exon. Exon length also varied, although intron length was shorter and more preserved. However, the order and quantity of exons and introns did not show a significant difference (Figure 6C). The protein sequences of *FoChi* genes from the *F. ox* and other fungi genomes were phylogenetically analyzed to investigate their evolutionary kinship. These *FoChi* genes were divided into four clades. In detail, there were eighteen genes in Clade I, including two homologs in *S. cerevisiae* genes and sixteen *FoChi* genes. There were eleven genes in Clade II, comprising seven *FoChi* genes and four homologs in *Trichoderma atroviride*. Twelve genes were classed into Clade III with one *FoChi* gene, six homologous genes in *Rhizophagus irregularis*, and three homologous genes from other fungi. The greatest number of genes were divided into Clade IV with twenty-two genes, with one FoChi gene (EXM26853) and twenty-one homologous genes from other fungi (Figure 6D).

Based on the RNA-seq results, the gene expression of twenty-five *FoChi* in root secretions was investigated and characterized into four groups in the pathogenicity of *F. ox*. The first group contained seven *FoChi* genes: *FoChi12*, *FoChi25*, *FoChi24*, *FoChi5*, *FoChi20*, *FoChi23*, and *FoChi21*. All seven genes were highly expressed in susceptible cotton root secretion co-cultures after 6 h, while their expression was low in resistant cotton root secretion co-cultures. It was assumed that these genes had an important effect on the pathogenicity of *F. ox*. The second group contained six *FoChi* genes: *FoChi13*, *FoChi9*, *FoChi6*, *FoChi17*, *FoChi19*, and *FoChi7*. Among them, *FoChi6*, *FoChi9*, and *FoChi13* genes were highly expressed in the co-culture of disease-resistant cotton root secretion for 12 h, and the expression levels of the genes were high in the co-culture of disease-resistant cotton root secretion, while the expression levels of the genes were low in the co-culture of disease-resistant cotton root secretion. *FoChi7*, *FoChi17*, and *FoChi19* genes had higher expression after 6 h of co-cultivation in disease-resistant cotton root secretions, while all had lower expression in susceptible cotton root secretion co-cultivation. The third group contained seven *FoChi* genes, *FoChi22*, *FoChi18*, *FoChi3*, *FoChi2*, *FoChi1*, *FoChi11*, and *FoChi4*, and the expression of these seven genes did not show any significant difference in the root secretions of susceptible cotton. The fourth group contained five *FoChi* genes: *FoChi15*, *FoChi8*, *FoChi16*, *FoChi14*, and *FoChi10*. Among them, *FoChi8*, *FoChi16*, *FoChi14*, and *FoChi10* genes had higher expression in the co-culture of disease-resistant cotton root secretions after 24 h and lower expression in the co-culture of susceptible cotton root secretions. However, there was no significant difference in the expression of the *FoChi15* gene in root secretions of disease-resistant and susceptible cotton (Figure 6E).

**Figure 7:**
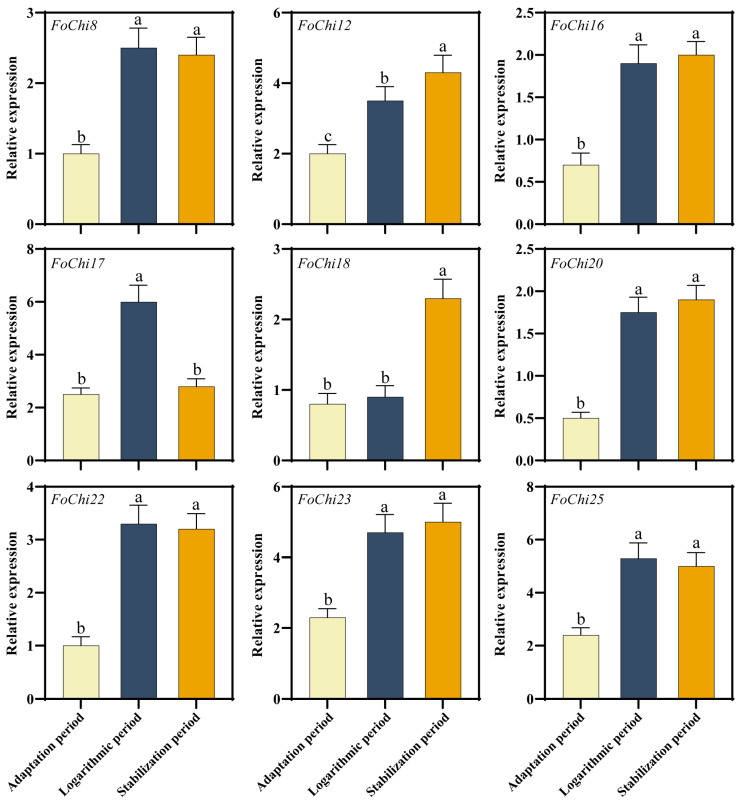
Transcriptional expression of nine *FoChi* genes at different developmental periods in *F. ox*. Vertical bars in each column represent ± SD of three replicates, and different letters represent significant differences (*p* < 0.05).

*FoChi* genes significantly expressed under water culture (the transcriptome data obtained by growing the *F. ox* under clear water conditions for 6, 12, 24, and 48 h) *F. ox* development (adaptation, logarithmic, and stabilization). The expression of most of the genes was significantly higher in the logarithmic and stabilization period than in the adaptation period, whereas the expression of *FoChi17* in the logarithmic period was significantly higher than that in the adaptation and stabilization period. Among them, *FoChi20* had the highest expression in the stabilization period, 3.4 times higher than that in the adaptation period (Figure 7). This indicated that the expression of *FoChi* in *F. ox* is period-differentiated. Therefore, the function of this gene in the pathogenicity mechanism of *F. ox* was further investigated in this study.

### 2.4. Silenced FoChi20 Gene Attenuated the Pathogenicity of F. ox and Enhanced Disease Resistance in Cotton

**Figure 8:**
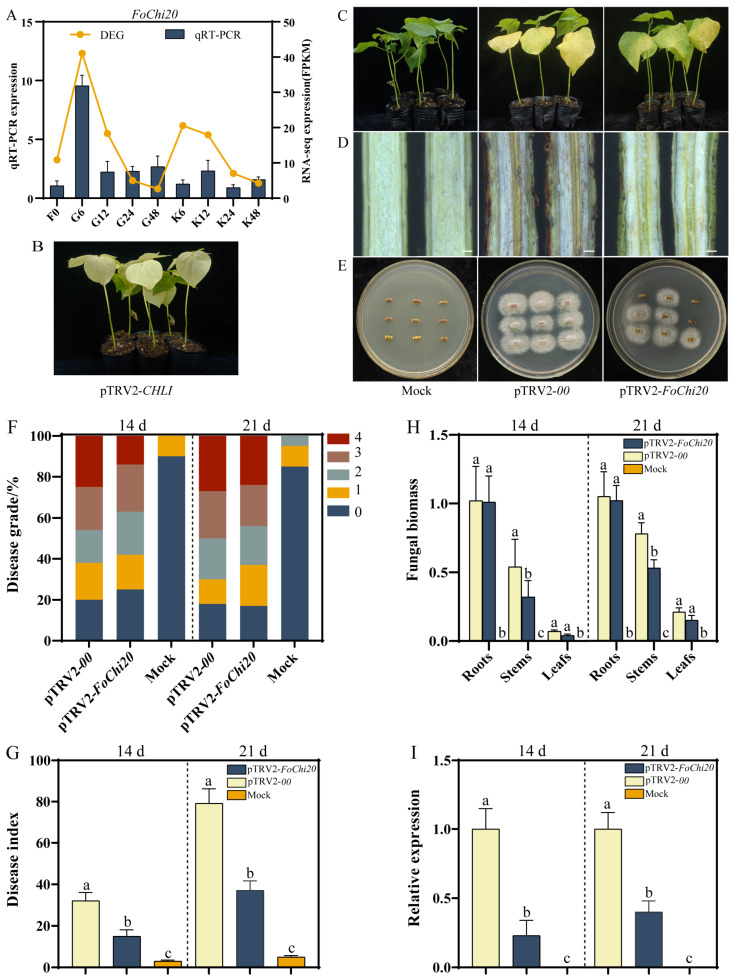
The expression pattern of *FoChi20* gene and HIGS assay results. (**A**) The expression pattern of *FoChi20* gene in the root secretion culture of susceptible varieties of cotton. F represents the treatment at 0 h. G represents the susceptible root secretion treatment for 6, 12, 24, and 48 h, respectively. K represents the resistant root secretion treatment for 6, 12, 24, and 48 h. (**B**) The cotton phenotype after 10 d of pTRV2-*CHLI*-treatment. (**C**) Cotton phenotypes 21 d after inoculation (Mock is water-treated, pTRV2-*00* is empty vector, pTRV2-*FoChi20* is the target gene silencing vector). (**D**) Stem phenotype after 14 d of inoculation. Bar = 0.3 mm. (**E**) Stem pathogen recovery culture 14 d after inoculation. (**F**) Cotton disease grade distribution. (**G**) Cotton disease index. (**H**) Relative fungal biomass in cotton. (**I**) The relative expression of target genes. Vertical bars in each column represent ± SD of three replicates, and different letters represent significant differences (*p* < 0.05).

Among the 25 *FoChi* genes, the main focus was on the *Fochi20* gene because it was closely associated with the pathogenicity of *F. ox* within the tan modules. In addition, the *FoChi20* gene was significantly upregulated in root secretion cultures of the susceptible variety when co-cultivated with root secretions of the susceptible variety for 6, 12, 24, and 48 h, with the highest expression in the 6 h treatment (Figure 8A), suggesting that the gene was able to respond to root secretions of the susceptible cultivar. Thus, the *FoChi20* was further selected as an essential candidate gene for silencing studies. After 10 d of injection, there was a whitening of true leaves in pTRV2-*CHLI*-treated plants (Figure 8B), indicating that the HIGS system could successfully suppress the expression of the target gene. A suspension of *F. ox* spores was used to inject cotton by the root wounding method. Plants inoculated with the control and pTRV2-*FoChi20* were evaluated for disease index at 14 d and 21 d post-inoculation. Fourteen days after inoculation, pTRV2-*00*-treated plants showed severe yellowing and wilting, whereas pTRV2-*FoChi20* plants showed only slight leaf yellowing. Twenty-one days after inoculation, leaf abscission of pTRV2-*00*-treated plants was even more severe, wilting started at the top, and the disease index of most plants was close to grade 4 with even total death. In contrast, pTRV2-*FoChi20*-treated plants only showed symptoms of leaf yellowing and wilting, with some leaf abscission, but the severity was significantly lower than that of pTRV2-*00*-treated plants (Figure 8C). Browning was less pronounced in plants treated with pTRV2-*FoChi20* than in plants treated with pTRV2-*00* (Figure 8D). A large number of *Fusarium spinosum* was isolated from the stem tissues of pTRV2-00-treated plants after recovery culture, whereas only a small amount of *F. ox* was isolated from the stem of pTRV2-*FoChi20*-treated plants (Figure 8E).

After 14 d of inoculation, pTRV2-*00*-treated plants had a higher degree of disease, with about 61% of cotton seedlings at level 2 and above and about 25% of cotton seedlings at level 4; after 21 d of inoculation, 28% of cotton seedlings were at level 4. However, the disease of pTRV2-*FoChi20*-treated plants was less severe, as about 42% of cotton seedlings were at the level of 0 and 1 after 14 d of inoculation. After 21 d of inoculation, about 30% of cotton seedlings were still at level 0 and 1, which was significantly lower compared to pTRV2-*00*-treated plants (Figure 8F). After 14 d of inoculation, the disease index of pTRV2-*FoChi20*-treated plants was 15.0, which was significantly lower than that of pTRV2-*00*-treated plants, which was 32.1. After 21 d of inoculation, pTRV2-*FoChi20*-treated plants had a disease index of 37.1, which was significantly lower than that of pTRV2-*00*-treated plants, which had a disease index of 79.2 (Figure 8G). After 14 and 21 d of inoculation, the stem fungal biomass of pTRV2-*FoChi20*-treated plants was significantly lower compared with that of pTRV2-*00*-treated plants (Figure 8H). Meanwhile, 14 and 21 d after inoculation, the expression of *FoChi20* in pTRV2-*FoChi20*-treated plants was significantly lower than that in pTRV2-*00*-treated plants, by 77% and 60%, respectively (Figure 8I), suggesting that the expression of *FoChi20* was suppressed in pTRV2-*FoChi20*-treated plants. All the results showed that the *FoChi20* gene could decrease the pathogenicity of *F. ox* and improve disease resistance in cotton.

**Figure 9:**
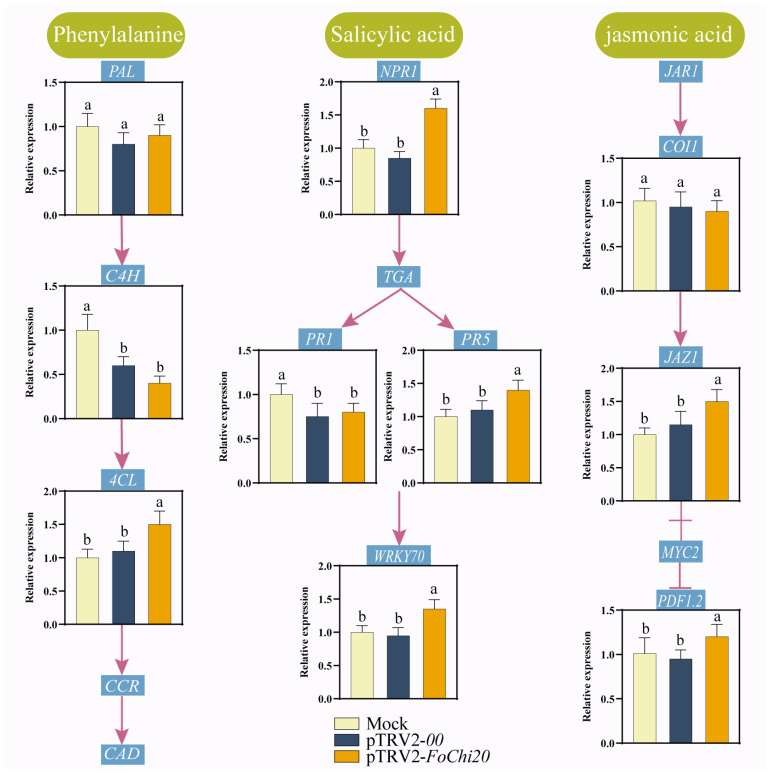
Expression of disease resistance-related genes. Vertical bars in each column represent ± SD of three replicates, and different letters represent significant differences (*p* < 0.05).

At 21 d after inoculation, the expression of *PAL*, *PR1*, and *COI 1* genes was not different in pTRV2-*FoChi20*-treated cotton compared with that of Mock and pTRV2-*00*-treated cotton, while the expression of *4CL*, *NPR1*, *PR5*, *WRKY70*, *JAZ1*, and *PDF1.2* genes was significantly up-regulated. This result indicates that the silencing *FoChi20* treatment of cotton by HIGS technology can activate the expression of cotton SA and JA pathway genes as well as other genes related to disease resistance, thus improving disease resistance in cotton (Figure 9).

## 3. Discussion

### 3.1. Root Secretions Could Be Widely Used to Inhibit F. ox Growth

Root secretions are a bridge between the plant, the soil, and soilborne pathogens, which can not only indirectly inhibit soilborne pathogens by changing the soil environment but also directly inhibit soilborne pathogens through their own actions [47,48,49]. Researchers used intercropping apple trees with *Allium cepa* and found that *Allium cepa* root secretions inhibited the growth of the mycelium and spore germination of *Fusarium oxysporum HBH 08*, thereby promoting the growth of apple seedlings [50]. In addition, the researchers found that root secretions from susceptible cucumber varieties promoted *F. ox* spore germination and growth, while root secretions from resistant varieties inhibited germination and growth, enhanced the resistance of cucumbers to wilt, reduced the incidence of cucumber wilt [51]. In this study, similar to the results of the above studies, the root secretion of the susceptible variety of Sea Island cotton could promote the growth of *F. ox;* the root secretion of the disease-resistant variety of Sea Island cotton could inhibit the growth of *F. ox*, suggesting that plant root secretions can affect the growth of *F. ox*, and the genes induced by them may play an important role in *F. ox* pathogenicity and host–pathogen interactions. It is speculated that there may be some substances in the root secretions of disease-sensitive varieties that inhibit the growth of pathogenic bacteria, which need to be further investigated.

### 3.2. Pathogenicity-Related Genes in F. ox Were Predicated

WGCNA was first applied to medical research and has begun to be increasingly applied to agricultural research in recent years, making important contributions to the cultivation of disease-resistant germplasm resources by mining disease-causing-related genes of plant pathogenic bacteria [52,53,54]. The researchers used WGCNA to analyze the transcriptome sequencing data of disease-resistant and disease-susceptible sugarcane varieties and obtained a total of 15 modules, four of which were significantly associated with black sigatoka resistance. A total of 38 hub genes were screened by calculating the gene connectivity in the corresponding networks. The results of this study provide gene targets for breeding sugarcane for black sigatoka resistance [55]. The important role of WGCNA analysis in pathogenicity-related gene mining was further confirmed. Therefore, in this study, WGCNA analysis identified two vital modules highly correlated with *F. ox* pathogenicity and screened out 10 related pivotal genes (Figure 3, Figure 4 and Figure 5), which will lay the foundation for the screening of *F. ox* pathogenicity genes in the later stage.

### 3.3. Chitinase Genes Influenced the Pathogenicity of Pathogenic Bacteria

Most fungal chitinases belong to the GH18 glycoside hydrolase superfamily and play roles in several growth periods, including cell wall degradation and modification. For example, spore germination, tip growth, mycelial maritimization, spore differentiation, autolysis, and reparasitism affect fungal growth and pathogenicity [56,57]. In addition, the deletion of the chitinase *MoChi1* in the filamentous fungus *M. oryzae* leads to slowed mycelial growth and reduced virulence, significantly reducing the pathogenicity of *M. oryzae* [58]. Similarly, a deletion mutation in the chitinase gene *NgCHIT-1* of *Neurospora crassa* resulted in reduced mycelial growth and spore germination, demonstrating that the chitinase gene effected mycelial cell wall remodeling and its pathogenicity [59]. All told, considering the important role of chitinase genes in fungal pathogenicity, we investigated the *F. ox* genome-wide *FoChi* gene family and found 25 *FoChi* genes (Figure 6 and Figure 7), among which the silencing of the *FoChi20* gene effectively reduced the pathogenicity of *F. ox* (Figure 8), and we believe that the *FoChi* genes will become a genetic resource for further research on the pathogenicity of *F. ox*.

### 3.4. The Value of HIGS Technology in Plant Disease Resistance Research

In recent years, HIGS technology is emerging using the principle of RNA interference, which targets and silences key genes of pathogenic fungi through the production of siRNAs in plant cells and widely used in plant–pathogen interactions [60,61]. Researchers also found that the *Ftf1* gene, a transcription factor of *F. oxysporum* f. sp. *cubense*, and the Velvet gene family can be used as HIGS target genes to significantly reduce the pathogenicity of the pathogen, and the transgenic banana plants created were significantly more resistant to wilt disease [62]. In the same vein, researchers silenced the chitinase genes *VdChs5* and *VdChs7* using HIGS technology, found deficiencies in cell wall integrity and virulence, significantly reduced pathogenicity of *V. dahliae*, and significantly enhanced resistance to wilt disease in cotton [63]. Therefore, in the present study, the silencing of the *FoChi20* gene by a HIGS assay (Figure 8) showed that the pathogenicity of *F. ox* was weakened and the resistance of cotton plants was enhanced, indicating that the chitinase *FoChi20* gene was closely related to the pathogenicity of *F. ox*, which confirmed the power of HIGS technology, playing a role in related studies.

## 4. Materials and Methods

### 4.1. Sample Collection of Cotton and Fusarium oxysporum *f. sp.* Vasinfectum

The resistant variety Xinhai 41 (XH-41) and susceptible variety Xinhai 14 (XH-14) were used to investigate the interaction between wilt and island cotton. The cotton plants were infected by injuring the roots when seedlings reached the two-leaf-one-heart stage, and each plant was inoculated with 20 mL of 1.0 × 10^6^ cfu·mL^−1^ spore suspension. The xylem of the cotton stalks were observed 21 days after the inoculation. The sensitivity to the disease was counted, and images of the sick phenotypes of the cotton were taken. The disease index was calculated as previous research published by Nowara [48]. The disease grade was classified as follows: 0 (no symptoms), 1 (0–25% wilted leaves), 2 (25–50% wilted leaves), 3 (50–75% wilted leaves), and 4 (75–100% wilted leaves). The disease index was calculated for each treatment according to the following formula: DI  = [(∑disease grades × number of infected plants)/(total checked plants × 4)] × 100. The fungus was recovered from infected cotton by surface sterilizing stem sections of infected cotton plants in 75% alcohol followed by 10% H_2_O_2_ for 60 min. The samples were rinsed three times with sterile water, placed on PDA medium, and cultured at 25 °C.

After being submerged in 75% alcohol for 10 min, cotton seeds were rinsed three times in distilled water. Seeds were then spread in a germination box with three layers of gauze to grow until dehulled, cotton roots were sponge-wrapped through a 35-hole foam board to prevent root damage, and finally, the foam board was placed in a plastic tub with five liters of Hogan’s nutrient solution while using an oxygen pump for continuous oxygenation. It took 45 days to gather a sample of root secretions. After calibration and filtering, the concentration was adjusted to 0.2 g·mL^−1^. Detailed information can be found in previous research published by Lou [46]. *F. ox* samples from 0 h were selected and labeled F0; *F. ox* from root secretion cultures of the susceptible variety XH-14, labeled G6, G12, G24, and G48; *F. ox* from root secretion cultures of the disease-resistant variety XH-41, labeled K6, K12, K24, and K48; and *F. ox* from water cultures, labeled W6, W12, W24, and W48.

### 4.2. F. ox Morphological and Molecular Biological Characterization

*F. ox* strain F327 was employed in this study. First, 200 μL of F327 spore liquid was taken onto the PDA medium evenly after 7 days of constant dark incubation at 25 °C. Using a 7 mm punch, a sufficient amount of mycelial mass was injected into Czapek’s medium, and the mixture was then shaken for 5–7 days in the dark at 28 °C 220 r·min^−1^. Four layers of gauze were used to remove the mycelium balls, and a hemocytometer plate was used to regulate the spore concentration to 1.0 × 10^6^ cfu·mL^−1^.

The 7 mm fungus cakes were put onto PDA medium and incubated at 25 °C in the dark for 7 days. The colony shape, color, and diameter of the colony were all inspected and measured with a straightedge. Following a sterile water wash, the mycelium and conidia were placed in conical flasks to be examined under a microscope for morphology.

We obtained the *Fov1-Eg* sequence from *F. ox* genome (Fusarium_oxysporum_f_sp_vasinfectum_25433_gca_000260175—Ensembl Genomes 59). Software primer 5.0 was used, and attention was taken to secondary structure prediction, inter-primer interaction analysis, and melting temperature for primers’ specific design. Cotton wilt-specific primers (*Fov1-Eg*) were obtained, the genomic DNA of the test strains was molecularly characterized by PCR (Table S1), and the genomic DNA of the pathogenic fungi was extracted using the CTAB technique. The PCR amplification reaction system was DNA 0.4 μL, PCR MIX (50 mmol·L^−1^) 1.0 μL, *Fov1-Eg*-F primer (10 μmol·L^−1^) 0.2 μL, *Fov1-Eg*-R primer (10 μmol·L^−1^) 0.2 μL, ddH_2_O 4.2 μL. The reaction procedure was 95 °C 5 min; 95 °C 30 s, 55 °C 30 s, 72 °C 90 s, 30 cycles; and 72 °C 2 min. PCR amplification products were detected by 1% agarose gel electrophoresis.

### 4.3. RT-qPCR Experiment

*F. ox* transcriptome analysis procedure was consistent with previous research published by Lou [46]. Ten DEGs were chosen for RT-qPCR validation to confirm the validity of RNA-seq results. By creating specific primers by Primer Premier and analyzing them on a Roche LightCycler^®^ 480 (Roche Diagnostics, Basel, Switzerland) using SYBR Green Mix (VazymE, Nanjing, China), the relative expression of the genes was calculated using the internal reference gene for the *F. ox* microtubulin gene (*β-tubulin*) (Table S1). All of the primers were created by Aricon Bio (Aricon Biotechnology Co., Ltd., Hangzhou, China). The reaction procedure was 94 °C 1 min, 94 °C 15 s, 58 °C 20 s, and 72 °C 20 s for 40 cycles, setting up 3 repetitions.

### 4.4. Identification of Chitinase Genes in F. ox

Fusarium_oxysporum.FO _Cotton_V1. GFF3, CDS, genome, and protein sequence files were downloaded from the Ensembl database website (https://fungi.ensembl.org/Fusarium_oxysporum_f_sp_vasinfectum_25433_gca_000260175/Info/Index, (accessed on 1 March 2024)). All of the chitinase genes in the GFF3 file were searched using the TBtools program (https://doi.org/10.1016/j.molp.2020.06.009, (accessed on 1 March 2024)) to find the *F. ox* chitinase genes initially. The key structural domains of the genes were further examined using the PFAM online software (http://pfam-legacy.xfam.org/, (accessed on 1 March 2024). EMBL-EBI, Cambridge, UK) for removing incomplete short pieces and repetitive sequences. Using the ExPASy-ProtParam program (https://web.expasy.org/protparam/, (accessed on 1 March 2024). Swiss Institute of Bioinformatics, Geneva, Switzerland), amino acid sequences were examined for protein residue number (aa), molecular weight (kD), and isoelectric point (pI). The online tool ProtComp 9.0 (http://www.softberry.com/berry.phtml?topic=protcomppl&group=programs&subgroup=proloc, (accessed on 1 March 2024). Softberry Company, Mount Kisco, NY, USA) was used to predict the subcellular localization of proteins.

### 4.5. Evolutionary Tree and Location of the Chitinase Genes

The structural domains of the chitinase genes were predicted by SMART. The structural domains of the protein were mapped using the TBtools software (https://doi.org/10.1016/j.molp.2020.06.009, (accessed on 1 March 2024)), which was also used to examine the number and location of exon–introns in the chitinase gene. Using NCBI BlastP (https://blast.ncbi.nlm.nih.gov/Blast.cgi (accessed on 1 March 2024). National Center for Biotechnology Information, Bethesda, MD, USA), the primary structural domains of 25 chitinase gene protein sequences were utilized as probe sequences for sequence comparison, and chitinase gene sequences from different species were chosen. ClustalW tool of MEGA 6.0 was employed to perform multiple sequence alignment for the protein sequences of chitinase genes from *F. ox* and other species. Next, a phylogenetic tree was created using the proximity linkage method.

### 4.6. Host-Induced FoChi20 Gene Silencing Assay

The expression of *FoChi* in different developmental stages of *F. ox* was collected from F327 strains in the acclimatization, logarithmic, and stabilization stages. The total RNA of each tissue was extracted and reverse transcribed into cDNA, which was analyzed by the RT-qPCR method mentioned above. To efficiently distinguish family genes, primers were designed in the UTR (Table S1).

The cDNA of F327 was used as a template to amplify the HIGS target fragment of the *FoChi20* gene with a fragment length of 438 bp, which was inserted into the pTRV2 vector, and the HIGS vector pTRV2-*FoChi20* was transformed into Agrobacterium tumefaciens GV3101 by electroexcitation method [63]. Agrobacterium tumefaciens GV3101 was transformed with pTRV1, pTRV2-*FoChi20*, the empty vector pTRV2-*00*, and the control vector pTRV2-*CHLI* by the electroshock transformation method [63]. When the two cotyledons of the cotton seedlings were spreading, a single colony of Agrobacterium tumefaciens containing pTRV1, pTRV2-*00*, pTRV2-*FoChi20*, and pTRV2-*CHLI* was picked and added to 30 mL LB liquid medium. An amount of 30 mL LB liquid medium and incubated overnight at 25 °C, 200 r/min. The shaking culture was put into a centrifuge at 4 °C for 10 min at 5000 r·min^−1^ to collect the bacterial bodies, add the spore suspension, and resuspend the bacterial solution to OD_600_ = 0.8. The prepared resuspensions containing pTRV2-*CHLI*, pTRV2-*FoChi20*, and pTRV2-*00* were mixed with the resuspension containing pTRV1 at a ratio of 1:1, respectively, and then injected with pTRV2-*CHLI* and pTRV2-*00*. The cotton was injected after 3 h of resting at room temperature. Cotton seedlings grown for 10 d were injected with a 1 mL needleless syringe using the compression method, and the bacterial solution was injected into the spreading cotyledons to fill the whole cotyledon. Eighty cotton seedlings were injected with pTRV2-*00* and pTRV2-*FoChi20* resuspensions, and the injected cotton was incubated in dark culture at 25 °C for 12 h and then returned to normal light culture.

After 7–10 d of Agrobacterium injection, when the true leaves of pTRV2-*CHLI*-treated cotton seedlings completely appeared yellow-white, they were inoculated with 1.0 × 10^6^ cfu/mL of spore suspension and infected with cotton susceptible cultivar XH-14 by the wounding method, and each plant was inoculated with 20 mL of spore suspension. Twenty pTRV2-*00*-treated cotton seedlings were selected as water-treated control (Mock), and the disease level rate and disease index of XH-14 were counted 15–30 d after infestation. Fourteen days after inoculation with *F. ox*, the roots, stems, and leaves of pTRV2-*00*- and pTRV2-*FoChi20*-treated cotton plants were collected, respectively, and total DNA was extracted, and RT-qPCR was performed using the cotton endogenous reference gene, *GhUBQ7* (DQ116441.1), and the blight-specific primers, *Fov1-Eg*-F and *Fov1-Eg*-R, to determine the *F. ox* bacterial biomass. Total RNA from cotton stems at 14 d after inoculation was extracted using a plant RNA extraction kit and reverse transcribed to cDNA, and the expression of *FoChi20* was analyzed using RT-qPCR with Tubulin as the internal reference gene and *FoChi20*-F and *FoChi20*-R as primers. At 14 d after inoculation, 10 cotton seedlings each of Mock, pTRV2-*00*, and pTRV2-*FoChi20* were randomly selected for the culm dissection test and *F. ox* recovery test to observe vascular browning and colony growth.

Total RNA was extracted and reverse transcribed into cDNA from cotton leaves inoculated with each treatment for 21 d. RT-qPCR was performed with the cotton disease resistance-related genes *PAL*, *C4H*, *4CL*, *COI1*, *JAZ1*, *PDF1.2*, *PR1*, *PR5*, *NPR1*, and *WRKY70*.

## 5. Conclusions

Two important modules, turquoise and tan, were obtained. Ten hub genes were screened using an interoperability network diagram, among which the chitinase *FoChi20* is a candidate gene related to the growth and pathogenicity of *F. ox*. A total of twenty-five *F. ox* chitinase genes (*FoChi1*-*FoChi25*) were identified from the *F. ox* genome and classified into four subgroups. Meanwhile, the *FoChi20* gene was able to respond to sensory cotton root secretions with the highest expression at 6 h of treatment. The silencing of the *FoChi20* gene by HIGS technology reduced the pathogenicity of *F. ox* and enhanced the disease resistance of cotton plants, providing a theoretical basis for the prevention and control of cotton blight.

## Data Availability

Data are contained within the article and Supplementary Material.

## Supplementary Materials

The following supporting information can be downloaded at https://www.mdpi.com/article/10.3390/ijms25158517/s1.
